# First-Principles Calculation of MoO_2_ and MoO_3_ Electronic and Optical Properties Compared with Experimental Data

**DOI:** 10.3390/nano13081319

**Published:** 2023-04-09

**Authors:** Eleonora Pavoni, Mircea Gabriel Modreanu, Elaheh Mohebbi, Davide Mencarelli, Pierluigi Stipa, Emiliano Laudadio, Luca Pierantoni

**Affiliations:** 1 Department of Materials, Environmental Sciences and Urban Planning, Marche Polytechnic University, Via Brecce Bianche, 60131 Ancona, Italy; e.pavoni@staff.univpm.it (E.P.); e.mohebbi@staff.univpm.it (E.M.); p.stipa@staff.univpm.it (P.S.); 2 Tyndall National Institute, University College Cork, T12 R5CP Cork, Ireland; mircea.modreanu@tyndall.ie; 3 Department of Information Engineering, Marche Polytechnic University, Via Brecce Bianche, 60131 Ancona, Italy; d.mencarelli@staff.univpm.it (D.M.); l.pierantoni@staff.univpm.it (L.P.)

**Keywords:** first-principles calculations, DFT, MoO_3_, MoO_2_, bandgap, optical spectra

## Abstract

MoO_3_ and MoO_2_ systems have attracted particular attention for many widespread applications thanks to their electronic and optical peculiarities; from the crystallographic point of view, MoO_3_ adopts a thermodynamically stable orthorhombic phase (α-MoO_3_) belonging to the space group *Pbmn*, while MoO_2_ assumes a monoclinic arrangement characterized by space group *P*2_1_/*c.* In the present paper, we investigated the electronic and optical properties of both MoO_3_ and MoO_2_ by using Density Functional Theory calculations, in particular, the Meta Generalized Gradient Approximation (MGGA) SCAN functional together with the PseudoDojo pseudopotential, which were used for the first time to obtain a deeper insight into the nature of different Mo–O bonds in these materials. The calculated density of states, the band gap, and the band structure were confirmed and validated by comparison with already available experimental results, while the optical properties were validated by recording optical spectra. Furthermore, the calculated band-gap energy value for the orthorhombic MoO_3_ showed the best match to the experimental value reported in the literature. All these findings suggest that the newly proposed theoretical techniques reproduce the experimental evidence of both MoO_2_ and MoO_3_ systems with high accuracy.

## 1. Introduction

The molybdenum oxides family includes compounds characterized by different Mo:O stoichiometries and polymorphs. Among them, the most common are MoO_3_ and MoO_2_, which differ in their chemical structure as well as in their electronic and optical properties [1,2]. Molybdenum oxides are widely used as redox-active catalysts in organic chemistry; for example, they act as catalysts in the oxidation reaction of methane or propane [3,4]. Moreover, the ability of the metal center to be involved in the redox process, together with the numerous oxidation states, and the four, five, or six coordination modes of molybdenum have attracted particular attention even for many other widespread applications such as sensors [5] and solar cells [6].

As for WO_3_ [7], MoO_3_ is also well known to have pronounced chromism—it is able to undergo color change under proper stimulations. Molybdenum oxide coloration can be determined by applying a potential (electrochromism) [8], by optical irradiation (photochromism) [9], and by changing the temperature (thermochromism) [10].

The doping of MoO_3_ with different elements and the creation of an oxygen vacancy inside the crystal lattice have enhanced the applicability of this material to electronic and optical devices. As a matter of fact, doping with special substituents allows for the controlling and tuning of the carrier concentration and band structure peculiarities [11,12]. Furthermore, optical devices with a reconfigurable response have recently attracted much attention, such as VO_2_ [13,14,15], which shows that a large modification in physical properties upon external input, even the reversible molybdenum oxides MoO_3_-to-MoO_2_ transformation associated with a dielectric-to-metallic character could be significant. Defect formation, vacancy presence, and dopant substitution seem to be also responsible for the ferromagnetic behavior in MoO_3_ [16,17,18,19] and the change in the crystal structure that may affect the electronic, optical, and mechanical properties of the material [20,21,22].

From the crystallographic point of view, MoO_3_ adopts a thermodynamically stable orthorhombic phase (α-MoO_3_) belonging to the space group *Pbmn* [2]. More, MoO_3_ can adopt a less stable phase, which is the monoclinic phase called β-MoO_3_ that spontaneously evolves into the orthorhombic structure at a temperature above 370 °C [23].

The orthorhombic polymorph is of special interest as it possesses a unique two-dimensional layered structure that is a fundamental requirement to be exfoliated in mono- or multiple-layered assemblies. As a matter of fact, after the discovery of graphene, other two-dimensional materials, such as metal chalcogenides and transition metal oxides, have attracted research interest and are considered appealing for a wide range of applications and the next generation of devices [24]. α-MoO_3_ is made by a series of bilayers oriented perpendicular to the [010] *y*-axis and kept together by non-covalent, mainly van der Waals interactions [24,25].

Regarding MoO_2_, it adopts a monoclinic crystallographic arrangement characterized by space group *P*2_1_/*c*. In MoO_2_, the Mo–O bond can be described as two different coordination environments, each one with three different Mo–O bond lengths [2]. Molybdenum dioxide is a metallic compound with interesting electronic properties.

MoO_2_ finds its major application in the organic chemistry field as a catalyst for isomerization and oxidation, and in the petrochemical industry for hydrodesulfurization, hydrogenation, and dehydrogenation reactions; it is a promising material for lithium-ion batteries [26] due to its high energy storage capacity and excellent electrochemical stability, and it is used as a substrate in surface-enhanced Raman spectroscopy (SERS) [27].

MoO_2_-based sensors are used for detecting hydrogen, oxygen, and carbon monoxide in different industries, and it is also used as a hole transport layer in organic and perovskite solar cells, which helps to improve their performance and stability; overall, MoO_2_ has significant potential for applications in various fields, and its use is expected to grow in the future [28].

In the present paper, we investigated the peculiar electronic and optical properties of both MoO_3_ and MoO_2_ by making use of Density Functional Theory (DFT) calculations and by comparing the obtained results with the experimental ones. For these reasons, among all the possible polymorphs of the molybdenum oxides, MoO_3_ and MoO_2_ were studied by considering the orthorhombic (α) and the monoclinic crystallographic structure, respectively, in order to reproduce the most thermodynamically stable phases. In detail, we used the newly proposed Meta Generalized Gradient Approximation (MGGA) exchange-correlation functional called Strongly Constrained and Appropriately Normed (SCAN) [29] together with the PseudoDojo pseudopotential [30]. The choice to use SCAN is that this approach adds the orbital kinetic energy density of each spin to the Generalized Gradient Approximation. In this way, SCAN can accurately predict different kinds of bonding, also including the effects of intermediate-range van der Waals interactions. SCAN is also fitted to additional appropriate norms, non-bonded systems such as atoms in which it can be accurate for the exchange and correlation energies separately, and not just for their sum as in bonded systems [31]. Since the interactions between Mo and O atoms can be ionic, single, and double covalent types, and this depends on the coordination, the number of oxygens, and the distances with centered Mo, the accuracy in the bond description is crucial for describing MoO_x_ systems. The semilocal density functional fulfills all known constraints that the exact density functional must satisfy. Studies have demonstrated that this functional is superior to most gradient-corrected functionals [32], and for these reasons, MGGA-SCAN was used to describe the MoO_3_ and MoO_2_ for the first time in this paper. To verify the reliability of the chosen method, the electronic properties of MoO_2_ and MoO_3_ were also calculated using the Generalized Gradient Approximation (GGA) PBEsol and hybrid HSE06 functionals. Both these methods are known to be very accurate for solid state oxide systems. In fact, both these materials have been already studied by the theoretical model with different methodologies. Authors such as Chen et al. [33], Rozzi et al. [34], and Eyert and coworkers [35,36] used the ab initio DFT based on local density approximation (LDA); Coquet and Willock [37] used the generalized gradient approximation (GGA) with the Hubbard correction term to understand the effect of oxygen vacancies while remarking on the importance of such a methodology for complex electronic systems. Scanlon et al. [2] used the generalized gradient approximation (GGA) with PBE in the plane wave basis set to study both MoO_3_ and MoO_2_. More recently, Gulomov et al. [38] used and compared two DFT approaches, namely, PBE and HSE06 functionals, calculating in both cases the band gap energy of MoO_3_ that was found to be, in the best case from HSE06 calculation, 3.027 eV.

The results obtained were confirmed and validated by comparison with literature data and our recorded experimental findings. In particular, the calculated density of the electronic state (DOS) was in good agreement with that in the literature, and the frontier orbitals detection, in terms of Highest Occupied Molecular Orbital (HOMO) and Lowest Unoccupied Molecular Orbitals (LUMO), confirmed the dielectric-to-conductor transition moving from MoO_3_ to MoO_2_. Moreover, the calculated optical spectra were compared with experimental findings, showing very good agreement; finally, the band-gap energy calculated for the dielectric α-MoO_3_ best matched the one reported experimentally rather than the other theoretical DFT-based studies. The use of our proposed computational method on Mo-based materials could clarify the interesting change in the properties of the systems, better describing the nature of each Mo–O bond in MoO_2_ and MoO_3_.

## 2. Materials and Methods

### 2.1. Theoretical Modeling and DFT Calculation

The Quantum Atomistic Toolkit (ATK) atomic-scale modeling platform was used to model all polymorphs and to perform all calculations [39]. The monoclinic MoO_2_ (*P2_1_/c*) and the orthorhombic MoO_3_ (*Pbmn*) polymorphs were modelled starting from the Materials Project database [40] and optimized. The electron basis was expanded in linear combination using the atomic orbital (LCAO) method for Mo and O entities resembling the SIESTA formalism [41]. In comparison with other basis sets, the whole electron LCAO calculations describe accurately electronic distributions both in the valence and the core region with a limited number of basis functions. All simulations were carried out using the MGGA density functional called SCAN for the electron exchange-correlation energy [42]. It is described as follows (1):

(1) Exc [n]=∫n(r)∈xc(n(r), |∇n(r)|,  t (r)) dr 

where *n* is the electron density, ∇n(r) is its gradient, while *t (r)* is the positive orbital kinetic energy density. This latter term is the additional one to the canonical GGA approach, and it is determined by (2):

(2) $$t\left(r\right)=\frac{1}{2}\sum_{i}^{Occ}{\left(\nabla \varphi_{i}\left(r\right)\right)}^{2}$$

where *φ_i_ (r)* are the Kohn–Sham orbitals.

For each atom, the ionic cores were represented by norm-conserving (NC) PDj pseudopotentials [30]. To model the systems, the periodic boundary conditions (PBCs) were used along all axes; in this way, it was possible to avoid problems with boundary effects caused by the finite size and to reduce the calculation time while maintaining high accuracy. The energy cut-off was fixed at 1200 eV, and the Brillouin-zone integration was performed over a 15 × 15 × 15 *k*-points grid.

The optical properties of the MoO_x_ structures were determined by two components of the dielectric function ε(ω) = ε_r_(ω) + *i*ε_i_(ω).

The imaginary part ε_i_ (ω) of the dielectric constant was determined from Equation (3) [43]:

(3) $$\varepsilon_{i}\left(\omega \right)=\frac{4\pi^{2}}{\Omega \omega^{2}}\underset{i\in \mathrm{HOMO},j\in \mathrm{LUMO}}{\sum }\underset{k}{\sum }W_{k}{\left|\rho_{ij}\right|}^{2}\delta \left(\varepsilon_{kj}-\varepsilon_{ki}-\hbar \omega \right)$$

where HOMO, LUMO, ω, Ω, *W_k_*, *ρ_ij_* were the valence band, conduction band, photon frequency, volume of the lattice, weight of the *k*-point, and elements of the dipole transition matrix, respectively.

The real part of the dielectric constant was obtained with Equation (4):

(4) $$\varepsilon_{r}\left(\omega \right)=1+\frac{1}{\pi }P\int_{0}^{\infty }d\bar{\omega }\frac{\bar{\omega }\varepsilon_{2}\left(\bar{\omega }\right)}{{\bar{\omega }}^{2}-\omega^{2}}$$

Finally, the refractive index (*n*) and extinction coefficient (*k*) of MoO_x_ systems were calculated as follows using Equations (5) and (6):

(5) $$\varepsilon_{r}\left(\omega \right)=n^{2}-k^{2}$$

(6) $$\varepsilon_{i}\left(\omega \right)=2nk$$

Finally, with the aim of comparing the simulated results between them and then to validate the computational approach, the electronic properties of MoO_2_ and MoO_3_ were calculated again using Generalized Gradient Approximation (GGA) PBEsol and hybrid HSE06 functionals, maintaining the same approach in relation to the basis sets and pseudopotentials adopted.

### 2.2. Experimental Section

Amorphous MoO_3_ and MoO_2_ thin films were deposited at room temperature by e-beam evaporation using MoO_3_ and MoO_2_ pellets (Pi-KEM 99.99% purity) in a Leybold SYRUS pro 710 on n-type silicon (100). The n-type Si (100) wafers were cleaned before the deposition using a standard RCA cleaning procedure. The nominal thickness of these MoO_3_ and MoO_2_ films targeted was 150 nm. After the deposition, MoO_3_ and MoO_2_ were annealed in N_2_ at 400 °C for 30 min in order to fully crystallize them.

Spectroscopic ellipsometry [44,45,46] was performed on the 150 nm-thick MoO_2_ film and on the 150 nm-thick MoO_3_ deposited on n-type silicon (100). The measurements were performed at a 70-degree incidence using a Woollam M2000 ellipsometer in the UV-VIS-NIR spectral range. The collected ellipsometry data were analyzed considering a four-layer optical model, i.e., air/MoO_3_/interfacial oxide/silicon, and for this purpose Woollam’s CompletEASE software was used.

## 3. Results and Discussion

### 3.1. Geometrical and Lattice Parameters

**Figure 1:**
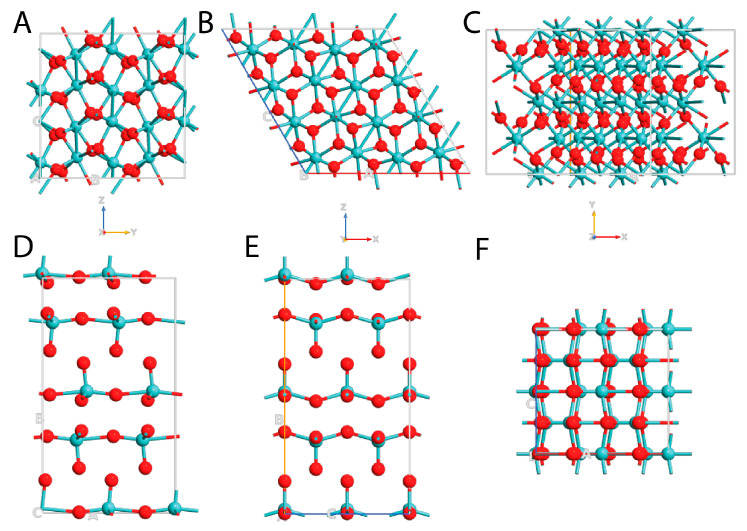
Schematic representation of all the examined systems: monoclinic MoO_2_ from *yz* plane (**A**), *xz* plane (**B**), and *xy* plane (**C**); orthorhombic MoO_3_ from *yz* plane (**D**), *xz* plane (**E**), and *xy* plane (**F**). Mo atoms are depicted in light blue and O in red.

In Figure 1 are reported the structures of both MoO_3_ and MoO_2_ from *xy*, *yz*, and *xz* planes, while in Table 1 are listed the values obtained after the geometry optimization for both the examined molybdenum oxides in terms of crystallographic lattice constant (the coordinate files of the optimized geometries are reported in the Supplementary Material). After the optimization of the geometry, the lattice constants of α-MoO_3_ were (a) 3.909 Å, (b) 13.855 Å, and (c) 3.681 Å, and α = β = γ = 90°. MoO_2_ adopted a monoclinic crystallographic arrangement characterized by space group *P*2_1_/*c*, with the lattice constant of (a) 5.625 Å, (b) 4.872 Å, and (c) 5.645 Å, and α = γ = 90°, and β = 120.5°. These values were in good agreement with the previous literature [2,47,48,49], confirming the ability of our methodology to reproduce both MoO_x_ systems.

**Figure 2:**
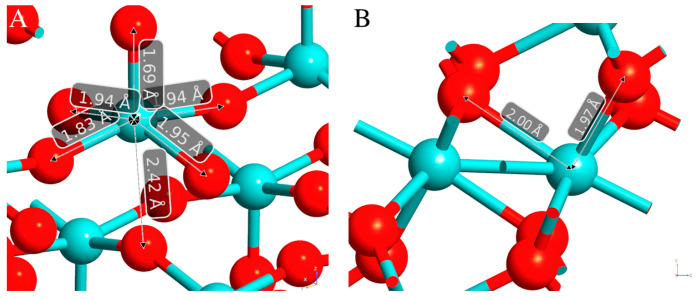
Bond lengths of MoO_3_ (**A**) and MoO_2_ (**B**). Mo atoms are depicted in light blue, while O are reported in red.

In more detail, α-MoO_3_ was made by distorted octahedra MoO_6_ with three different crystallographic oxygens: (i) single-coordinated O(I) bonded only to one Mo atom (Mo–O length of 1.67 Å), (*ii*) two-coordinate O(II) located symmetrically to two Mo atoms (Mo–O lengths of 1.74 Å and 2.25 Å), and (*iii*) three-coordinate O(III) oxygens in which two were symmetrically positioned between two Mo atoms (Mo–O lengths of 1.94 Å) and the third one interacted with Mo on the other layer (length of 2.33 Å) (Figure 2A). In MoO_2_, the Mo–O bond could be described as two different coordination environments with Mo–O bond lengths of about 2.00 Å and 1.97 Å (Figure 2B).

### 3.2. Band Structure, Band Gap, and Density of the Electronic State

**Figure 3:**
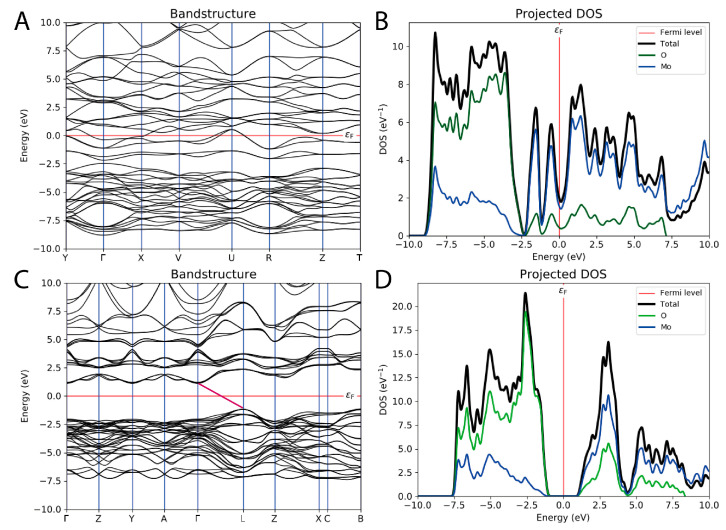
Band structure (**A**) and projected density of electronic states (**B**) of monoclinic MoO_2_. Band structure (**C**) and projected density of electronic states (**D**) of orthorhombic MoO_3_. The Fermi level is depicted as a red line, and the bandgap of MoO_3_ is evidenced as a pink line.

In Figure 3 are reported the band structure and the PDOS of both MoO_2_ and α-MoO_3_. The different oxidation states of molybdenum, which are +4 in MoO_2_ and +6 in MoO_3_, and the crystallographic displacement of the atoms, monoclinic for MoO_2_ and orthorhombic for MoO_3_, led to completely different electronic behaviors. As attested by the band structure (Figure 3A), MoO_2_ had zero band gap energy and the valence and the conduction bands were overlapping at the Fermi level. The DOS showed how the main contribution to the valence band originated from the oxygen, even if near the Fermi level the situation reversed and molybdenum started to play a fundamental role. The portion between −8 eV and −2.5 eV was mainly composed of the O 2^p^ orbitals, and only a minor contribution arose from the d states of Mo that by contrast became predominant between –2.5 eV and the Fermi level. Above the Fermi level, the Mo 4^d^ states determined the main trend with only a small involvement of the O states. Mo resulted in the leading responsibility of the conduction band, while above the Fermi level the 4^d^ states of Mo determined the main trend with only a small contribution of the O states (Figure 3B). These results perfectly match what was observed by other authors previously [35,36].

MoO_3_ is an indirect bandgap material; thus, the energy band gap resulted in 3.16 eV (with a direct band gap of 2.27 eV) (Figure 3C), and, to the best of our knowledge, this value is the one that best matches the experimental one of 3.2 eV [12], also considering other theoretical studies [2], where a band gap of 3.027 eV was found with the HSE06 method [38]. Similarly to the MoO_2_, even for MoO_3_ the PDOS calculation showed that the valence band derived from the oxygen 2^p^ states with a small contribution of the d state of Mo. Above the Fermi level, in the conduction portion, the Mo 6^d^ states determined the principal trend with a minor involvement of the O states (Figure 3D). These results are in line with previous literature reports [2].

**Figure 4:**
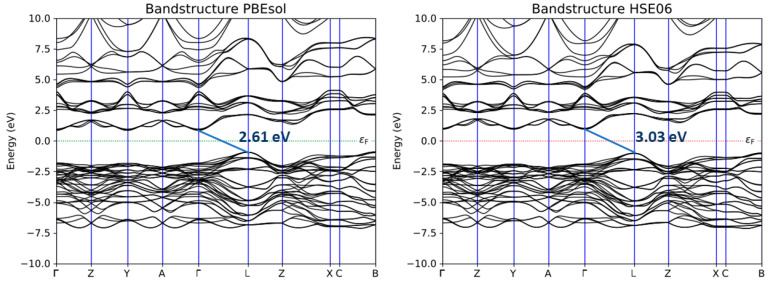
Band structure of MoO_3_ calculated with PBEsol and HSE06 functionals.

With the aim of testing the effective reliability of the SCAN functional, the MoO_3_ band structure was calculated again using the GGA PBEsol and the hybrid HSE06 functionals (Figure 4). The results were compared with the those obtained with the MGGA approach. The indirect bandgap detected with PBEsol was 2.61 eV, which means that the Generalized Gradient Approximation tended to underestimate the energy gap between valence and conduction bands. Using HSE06, the incorporation of a portion of the exact exchange from Hartree–Fock theory allowed us to obtain an indirect bandgap value of 3.03 eV, which is also in line with other previously conducted studies [38]. In any case, the SCAN functional was found to be the most accurate for the prediction of electrical properties of Mo–O-based systems, and for this reason, all the next calculations reported were performed using this MGGA approach.

**Figure 5:**
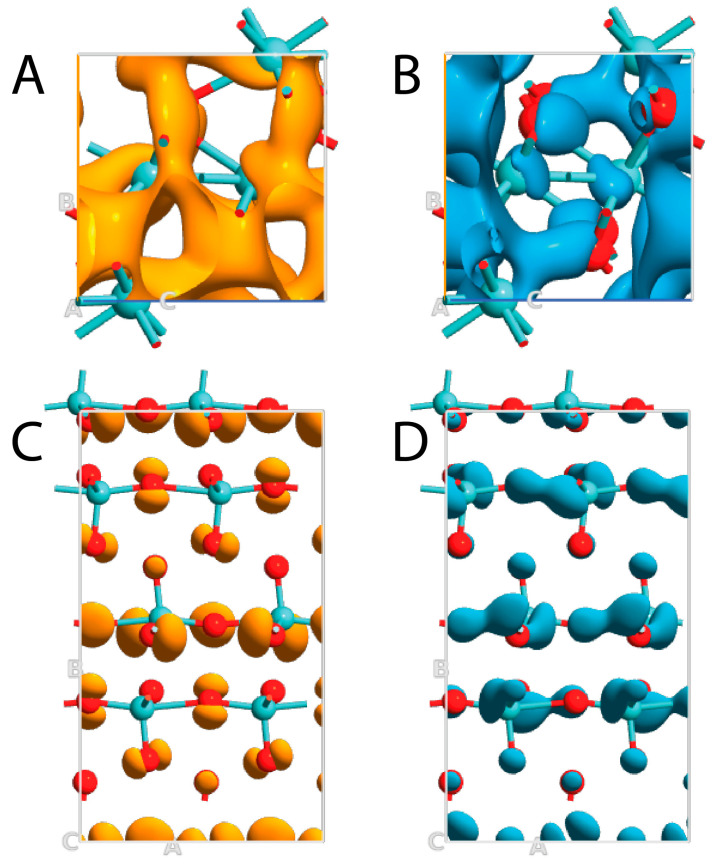
HOMO–LUMO representation of MoO_2_ (**A**,**B**) and MoO_3_ (**C**,**D**). HOMO and LUMO are reported in orange and blue, respectively.

The HOMO–LUMO visualization of both MoO_x_ materials was also reported to better indicate the metallic and dielectric behaviors of MoO_2_ and MoO_3_, respectively. Starting with the first one, LUMO was evidently present in correspondence of the Mo atoms, even if it was possible to easily identify it also on the O atoms in a symmetrical manner by following the space group of the unit cell (Figure 5A). This confirmed that the largest contribution in the bands beyond the Fermi level came from the 4^d^ electrons of Mo, and only a small contribution was associated with the 2^p^ electrons of O. In a parallel way, HOMO followed a symmetric trend showing a higher localization on O atoms and moving again with a lower contribution on Mo entities. This behavior is in perfect agreement with the DOS plot, since the highest contribution in the bands below the Fermi level arose from the 2^p^ electrons of O, and only a very small participation was attributed to the 4^d^ electrons of Mo (Figure 5B). In any case, HOMO and LUMO clouds complemented each other in the MoO_2_ structure, confirming the metallic behavior and the bonding homology between Mo and O entities.

Focusing on MoO_3_, peculiar characteristics were detected. In this case, LUMO was again predominantly localized on Mo entities, but only axial O atoms showed a small contribution (Figure 5C). This means that the Mo–O connections exhibited both ionic (the charge transfer from 2^p^ orbital of oxygen to molybdenum) and covalent (charge accumulation in the region of Mo–O) components, and the MoO_3_ bonds were not equal. Furthermore, HOMO was particularly localized on the O atoms in an asymmetric way (Figure 5D). This means that the valence band came from the 2^p^ electrons of O and only in a small part from 4^d^ electrons of Mo, while the opposite was observed for the conduction bands.

### 3.3. Experimental and Theoretical Optical Spectra

In order to confirm the ability of the DFT methodology proposed herein to describe the peculiarities of both MoO_2_ and MoO_3_, the experimentally recorded optical spectra were compared to the simulated ones. The evaluation regarded (*i*) the refractive index, which is useful to understand the ability of the matter to bend or refract the light that passes through the material itself; (*ii*) the extinction coefficient, which represents the capability of the matter to absorb the light; (*iii*) the real part (ε_r_) of the dielectric function, which describes the ability of the matter to interact with an electric field without absorbing energy; and (*iv*) the imaginary part (ε_i_) of the dielectric function, which describes the ability of the matter to permanently absorb energy from a time-varying electric field; the spectra were reported in the function of the energy of the applied (and simulated) electric field expressed in eV.

**Figure 6:**
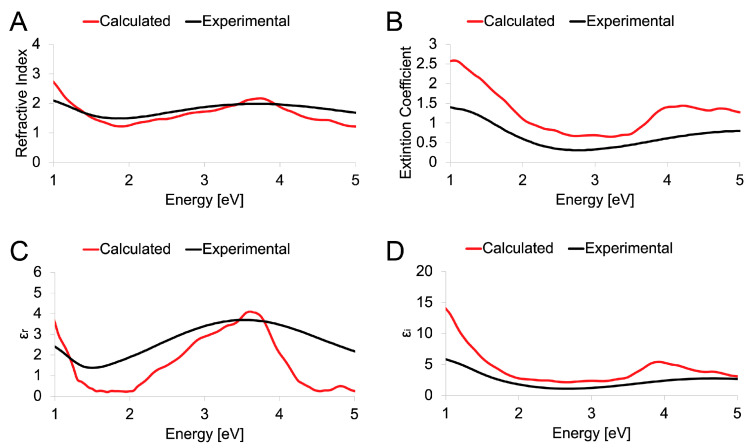
Calculated (red) and experimental (black) optical spectra of monoclinic MoO_2_. Refractive index (**A**), extinction coefficient (**B**), real part ε_r_ (**C**), and imaginary part ε_i_ (**D**) of the dielectric constant reported as a function of the energy.

The MoO_2_ optical spectra, simulated and recorded experimentally, are reported in Figure 6. From the comparison between the theoretical curve (in red) and the experimental (black) it is possible to notice good agreement in all four reported cases, with a small overestimation in the calculated spectra in terms of the extinction coefficient and the imaginary part of the dielectric constant. The reason for the small discrepancies between calculated and experimental evidence may be due to some small differences in the three-dimensional systems. In fact, MoO_2_ and MoO_3_ were considered in simulations as single crystals, while polycrystalline structures can be obtained during fabrication. These differences in microstructures of the materials were reflected in the optical properties observed and plotted together. Overall, good agreement was observed in the position of most critical points in the optical constants spectra. Moreover, when a disorder occurred, a change in the magnitude of the optical properties was expected.

**Figure 7:**
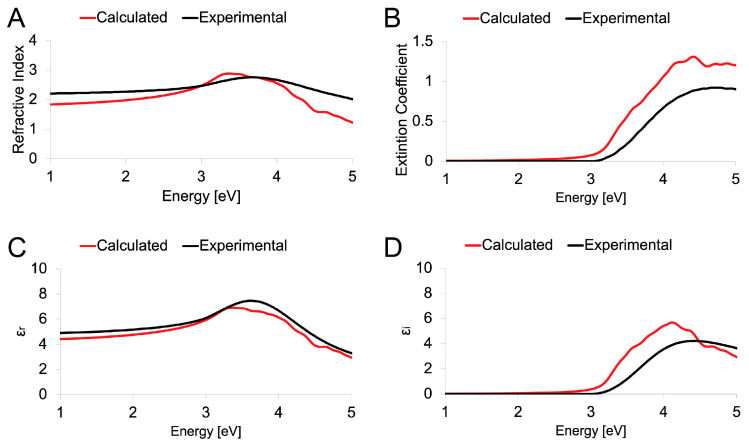
Calculated (red) and Experimental (black) optical spectra of orthorhombic MoO_3_. Refractive index (**A**), extinction coefficient (**B**), real part ε_r_ (**C**), and imaginary part ε_i_ (**D**) of the dielectric constant reported as a function of the energy.

Similarly, Figure 7 reported the optical spectra of MoO_3_. In this case, agreement between the experimental obtained and the estimated by the theoretical method was more evident, demonstrating the capability of the MGGA-SCAN proposed methodology to predict and verify the experimental findings.

The slight increase in discrepancy between experimental and simulated data for MoO_2_ can be attributed to the metallic character of the material, since this behavior is more difficult to reproduce with first-principle methods during optical properties calculations. Nevertheless, the SCAN functional seems to satisfactorily approach the experimental evidence.

## 4. Conclusions

In the present study, the properties of the well-known MoO_3_ and MoO_2_ systems were investigated. These compounds have attracted the attention of the scientific community thanks to their electronic and optical properties. MoO_3_ assumes an orthorhombic phase, named α-MoO_3_ that belongs to the space group *Pbmn*; MoO_2_ adopts a monoclinic crystallographic disposition described by space group *P*2_1_/*c.* The electronic and optical properties of both MoO_3_ and MoO_2_ were investigated using the MGGA-SCAN functional and the PseudoDojo pseudopotential, and then our calculated results were compared with previously reported experimental data and our recorded optical spectra. The results obtained confirmed that the chosen theoretical modeling methodology is highly accurate and able to reproduce the experimental findings of both MoO_3_ and MoO_2_. Moreover, it is important to underline that the band structure and the respective band gap calculated for MoO_3_ is the one that best matches the experimental one. Even by repeating the calculation with other known and widely used functionals, it was not possible to obtain the optimal bandgap, thus indicating the high sensitivity of the chosen method. The HOMO–LUMO descriptions of both MoO_2_ and MoO_3_ better clarified the peculiarities of these materials, shedding light on the role of different Mo–O bonds on the basis of metallic–dielectric behavior. Additionally, the recorded optical spectra in terms of refractive index, extinction coefficient, and the real and imaginary parts of the dielectric constant were in very good agreement with the corresponding calculated values by means of our *ab initio* methodology. The adopted first-principles study verified the experimental data available, identified the effects of one more O atom in the Mo-based structure, and provided a reasonable prediction of the physical–chemical properties of both systems, allowing us to clarify in detail the properties of these materials at the nanoscale.

## Figures and Tables

**Table 1:** Comparison between our theoretical results and experimental lattice vectors [47,48] for monoclinic *P2_1_/c* MoO_2_ and orthorhombic *Pca2_1_* MoO_3_.

		This Work	Experimental		This Work
Orthorhombic MoO_3_	a	3.909 Å	3.962 Å	α	90°
	b	13.855 Å	13.855 Å	β	90°
	c	3.681 Å	3.699 Å	γ	90°
Monoclinic MoO_2_	a	5.625 Å	5.611 Å	α	90°
	b	4.872 Å	4.856 Å	β	120.5°
	c	5.645 Å	5.628 Å	γ	90°

## Data Availability

Not applicable.

## Supplementary Materials

The supporting information about the input geometries of both the Mo systems can be downloaded at: https://www.mdpi.com/article/10.3390/nano13081319/s1.
