# Supplementary material for: First-Principles Calculation of MoO2 and MoO3 Electronic and Optical Properties Compared with Experimental Data
Source: Nanomaterials (Basel). 2023 Apr 9;13(8):1319. doi: 10.3390/nano13081319 (PMC10144520; doi:10.3390/nano13081319)
Supplement: Supplementary file 1 [file nanomaterials-13-01319-s001.zip › nanomaterials-2345979-supplementary.pdf]

Supplementary Materials

First-Principles Calculation of MoO_2 and MoO_3 Electronic and Optical Properties Compared with Experimental Data

Eleonora Pavoni [1], Mircea Gabriel Modreanu [2], Elaheh Mohebbi [1], Davide Mencarelli [3], Pierluigi Stipa [1], Emiliano Laudadio [1,*] and Luca Pierantoni [3]

[1] Department of Materials, Environmental Sciences and Urban Planning, Marche Polytechnic University, Via Brecce Bianche, 60131 Ancona, Italy; e.pavoni@staff.univpm.it (E.P.); e.mohebbi@staff.univpm.it (E.M.); p.stipa@staff.univpm.it (P.S.)
[2] Tyndall National Institute, University College Cork, T12 R5CP Cork, Ireland; mircea.modreanu@tyndall.ie
[3] Department of Information Engineering, Marche Polytechnic University, Via Brecce Bianche, 60131 Ancona, Italy; d.mencarelli@staff.univpm.it (D.M.); l.pierantoni@staff.univpm.it (L.P.)
* Correspondence: e.laudadio@staff.univpm.it

Input geometry of MoO_2

```
# Set up lattice

lattice = Monoclinic (5.54483*Angstrom, 4.87254*Angstrom, 5.64537*Angstrom, 90.0*Degrees, 120.5*Degrees, 90.0*Degrees)


# Define elements
elements = [Molybdenum, Molybdenum, Molybdenum, Molybdenum, Oxygen, Oxygen,
        Oxygen, Oxygen, Oxygen, Oxygen, Oxygen, Oxygen]

# Define coordinates
fractional_coordinates = [[ 0.229730913483,  0.007816652727,  0.213086875905],
                          [ 0.229728221644,  0.491773196715,  0.712731710353],
                          [ 0.769542910678,  0.991363046157,  0.786202773004],
                          [ 0.769545602517,  0.507406502169,  0.286557948549],
                          [ 0.111701453446,  0.718438490275,  0.377443761819],
                          [ 0.111698761607,  0.780331058051,  0.877088586274],
                          [ 0.887575062554,  0.218848640833,  0.122201062636],
                          [ 0.887572370715,  0.280741208609,  0.62184588709 ],
                          [ 0.390349418272,  0.197725282573,  0.591309054382],
                          [ 0.390352110111,  0.301864566869,  0.091664229928],
                          [ 0.60892171405 ,  0.697315132015,  0.907625418981],
                          [ 0.608924405889,  0.801454416311,  0.407980594527]]
```

Input geometry of MoO_3

```
# Set up lattice
lattice = Orthorhombic (3.909*Angstrom, 13.855*Angstrom, 3.68131*Angstrom, 90.0*Degrees,
90.0*Degrees, 90.0*Degrees)


# Define elements
elements = [Oxygen, Oxygen, Molybdenum, Oxygen, Oxygen, Oxygen, Oxygen,
        Molybdenum, Oxygen, Oxygen, Molybdenum, Oxygen, Oxygen, Oxygen,
        Oxygen, Molybdenum]

# Define coordinates
fractional_coordinates = [[ 0.037785290332,  0.137982542827,  0.000000227662],
                  [ 0.548288252624,  0.189282496069,  0.500000257704],
                  [ 0.507387648141,  0.309244274572,  0.500000320919],
                  [ 0.06540607799 ,  0.324759633975,  0.50000025144 ],
                  [ 0.585348864739,  0.34763758581 ,  0.000000262578],
                  [ 0.584659258008,  0.479104281039,  0.500000217995],
                  [ 0.104682870799,  0.502068181388,  0.000000208718],
                  [ 0.66198538547 ,  0.5173553747  ,  0.000000189186],
                  [ 0.615418711333,  0.637239874338,  0.000000241267],
                  [ 0.106979591451,  0.690072960965,  0.500000226912],
                  [ 0.147767493255,  0.810064791932,  0.500000128325],
                  [ 0.589546057794,  0.826275592252,  0.50000019184 ],
                  [ 0.069135486408,  0.848324778092,  0.000000174242],
                  [ 0.068472360777,  0.979844330377,  0.500000216522],
                  [ 0.548313826659,  1.002687042621,  0.000000229065],
                  [-0.00933012138 ,  1.018047094926,  0.000000176113]]
```
